# Vancomycin-associated hemorrhagic occlusive retinal vasculitis masquerading as central retinal vein occlusion

**DOI:** 10.5935/0004-2749.2022-0132

**Published:** 2023-03-08

**Authors:** Virginia Mares, Carlos Eduardo dos Reis Veloso, Marcio Bittar Nehemy

**Affiliations:** 1 Department of Ophthalmology, Universidade Federal de Minas Gerais, Belo Horizonte, MG, Brazil

**Keywords:** Antibiotic prophylaxis, Cataract removal, Drug hy-persensitivity, Macular edema, Retinal vasculitis, Extração de catarata, Antibioticoprofilaxia, Hiper-sensibilidade a drogas, Edema macular, Vasculite retiniana

## Abstract

A 69-year-old female was referred with sudden unilateral painless decreased vision that began 2 days after uncomplicated cataract surgery in the left eye. Visual acuity was hand motion and biomicroscopy showed a mild anterior chamber reaction, no hypopyon, and an intraocular lens that had been placed within the capsular bag. A dilated fundus examination revealed optic disk edema, widespread deep and superficial intraretinal hemorrhages, retinal ischemia, and macular edema. A cardiological evaluation was normal and thrombophilia tests were negative. After surgery, prophylactic vancomycin (1mg/0.1ml) had been injected intracamerally. The patient was diagnosed with hemorrhagic occlusive retinal vasculitis likely secondary to vancomycin hypersensitivity. Recognition of this entity is important to ensure early treatment and the use of intracameral vancomycin in the fellow eye should be avoided after cataract surgery.

## Abstract (pt)

Esse caso se refere a uma paciente de 69 anos, sexo feminino, com relato de baixa acuidade visual súbita e indolor no olho esquerdo, de início 2 dias após cirurgia de catarata sem complicações. A acuidade visual era de movimento de mãos e a biomicroscopia mostrou reação de câmara anterior moderada, sem hipópio, e lente intraocular posicionada dentro do saco capsular. A fundoscopia evidenciou edema de disco óptico, hemorragias difusas intrarretinianas superficiais e profundas, isquemia retiniana e edema macular. A avaliação cardiológica foi normal e os testes para trombofilia foram negativos. Ao final da cirurgia foi injetado antibioticoprofilaxia com vancomicina (1mg/0,1ml) na câmara anterior. A paciente foi diagnosticada com vasculite hemorrágica oclusiva da retina secundária à hipersensibilidade a vancomicina. O reconhecimento dessa entidade é importante para o tratamento precoce e para evitar o uso de vancomicina intracameral em caso de cirurgia de catarata no olho contralateral.

## INTRODUCTION

Cataract removal is one of the most commonly performed surgeries in the world and bacterial endophthalmitis is a potentially devastating complication of this procedure, with an incidence rate of around 0.1%^(1)^. In 2007, a randomized prospective study conducted by The European Society for Cataract and Refractive Surgery (ESCRS) reported the benefits of direct intracameral injection of cefuroxime (1mg/0.1ml) to prevent bacterial endophthalmitis after cataract surgery^(2)^. Since then, many studies have demonstrated the efficacy and safety of this prophylaxis, including an ESCRS guideline in 2013^(3)^. A survey by the American Society of Cataract and Refractive Surgery (ASCRS) found that the percentage of surgeons using intracameral antibiotics after cataract surgery increased from 30% in 2007 to 50% in 2014^(4)^. Although the ESCRS study was carried out with cefuroxime, vancomycin is one of the most commonly used antibiotics for endophthalmitis prophylaxis be-cause of its safety, availability, and efficacy^(5^-^7)^. However, in the past few years, hemorrhagic occlusive retinal vasculitis (HORV), a rare and dramatic retinal complication related to the intraocular use of vancomycin has been identified^(6^,^8)^. Herein, we present a case of unilateral HORV, initially misdiagnosed as central retinal vein occlusion (CRVO).

## CASE REPORT

**Figure 1:**
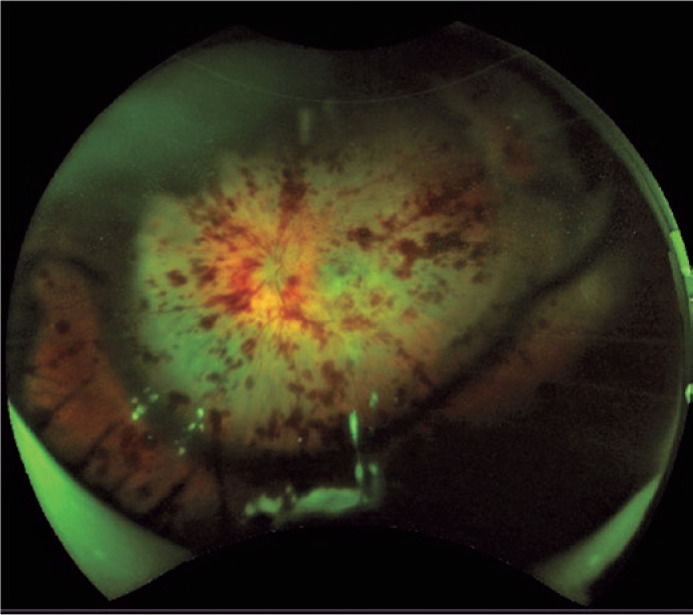
A pan-retinal fundus image of the eye of a patient with hemorrhagic occlusive retinal vasculitis showing edema of the optic disc, widespread deep and superficial hemorrhages, and macular edema.

**Figure 2:**
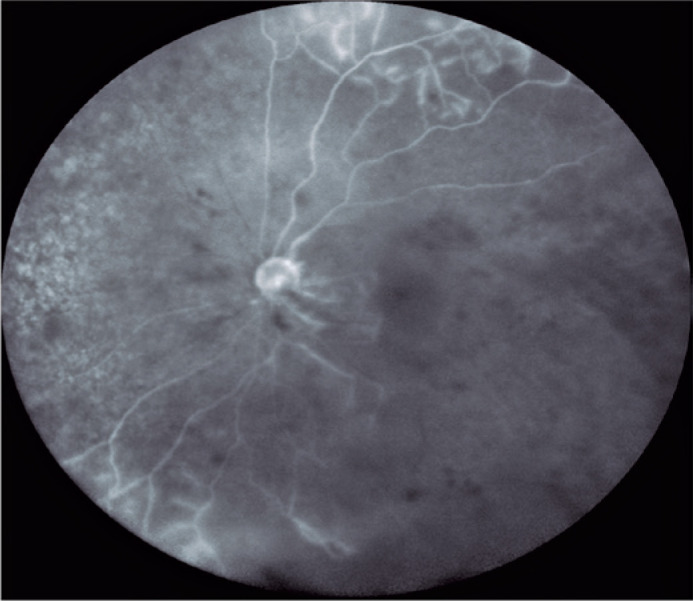
Fluorescein angiography image from a patient with hemorrhagic occlusive retinal vasculitis showing peripheral vasculitis and the near--complete absence of retinal perfusion, including the macula.

**Figure 3:**
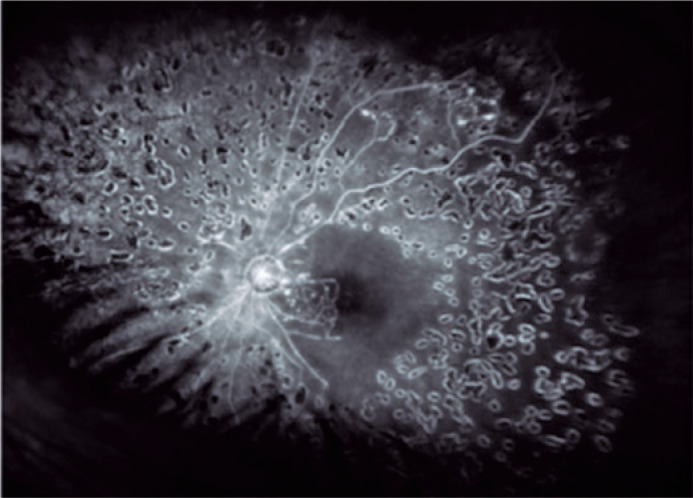
Fluorescein angiography image from a patient with hemorrhagic occlusive retinal vasculitis showing pan-retinal photocoagulation and macular ischemia.

A 69-year-old female presented with painless blurred vision 2 days after uncomplicated cataract surgery in the left eye. A normal un-dilated examination reported normal results on the first postoperative day. The patient was initially misdiagnosed with CRVO and one injection of bevacizumab (1.25mg/ 0.05ml) was administered before the patient was referred to our institution. On her first visit to the Instituto da Visão, Belo Horizonte, Brazil, 7 days after the cataract surgery, the patient’s visual acuity in the left eye was hand motion. Biomicroscopy showed a mild anterior chamber reaction and an intraocular lens that had been placed within the capsular bag. A fundus examination of the left eye showed optic disk edema, widespread deep and superficial hemorrhages, and macular edema (Figure 1). The right eye examination was unremarkable. The cataract surgeon reported that he had used vancomycin (1mg/0.1ml) for intracameral antibiotic prophylaxis at the end of the surgery. A cardiological evaluation and hematological thrombophilia screening were unremarkable. Fluorescein angiography showed extensive areas of nonperfusion of the retina (Figure 2) and optical coherence tomography showed macular edema. The patient was diagnosed with HORV, likely secondary to vancomycin hypersensitivity, and started on oral prednison 60 mg per day followed by gradual reduction. We also administered 4 intravitreal injections of bevacizumab (1.25mg/0.05ml) and 1 intravitreal injection of triamcinolone, pan-retinal photocoagulation, and 8 weeks of topical hypotensive medications during the first-year follow-up. Despite this intensive treatment, the condition evolved into neovascular glaucoma one year later, and visual acuity remained hand motion (Figure 3).

## DISCUSSION

Endophthalmitis is one of the complications most fea-red by cataract surgeons. Prophylaxis with intracameral antibiotics has been shown an effective and safe means of decreasing the incidence of endophthalmitis^(2^,^3^,^5)^, and vancomycin is one of the antibiotics most used for this purpose, with safe and satisfactory results^(5^,^9)^. Despite this research evidence and its widespread adoption by many surgeons around the world, the off-label use of prophylactic intracameral antibiotics during cataract surgery is still controversial. It is associated with increa-sed costs, dilution errors, and contaminants that may increase the risks to patients. In addition, it may contribute to the emergence of drug-resistant organisms^(10)^.

HORV secondary to vancomycin hypersensitivity is extremely rare and its exact prevalence is unknown. It occurs after intraocular procedures, however; its onset is delayed and patients usually seek treatment 1-21 days after surgery. In most cases, visual acuity is less than 20/40 at initial presentation. Occasionally, HORV may not cause significant early symptoms and can only be detected by a dilated retinal examination. Treatment outcomes are usually poor and a large retrospective study found that 22% of cases present with no light perception, and 61% attain a final visual acuity of 20/200 or worse^(7)^. Our patient presented at our institute with decreased vision 2 days after surgery, at which time, her visual acuity was hand motion. We believe her profound vision loss was mainly due to the extensive retinal ischemia, which involved the macular region. The fluorescein angiography images of our case shown in Figures 2 and 3 highlight the areas of vascular occlusion. There is often venular staining or leakage at the border of the retinal vascular occlusion and the optic nerve can be hyperfluorescent in later phases of the examination. Optical coherence tomography may show hyperreflectivity and thickening of the inner retinal layers, as well as intraretinal cysts^(7)^. The differential diagnosis of HORV includes acute postoperative endophthalmitis, viral retinitis, medication toxicity, and CRVO^(7)^. The similarities between these conditions make this a challenging diagnosis. Our case was initially misdiagnosed as CRVO before she was referred to our institution. To exclude any possible underlying conditions, we performed a systemic work-up on our patient. The pathogenesis of intravascular thrombosis in HORV is still unknown but its presentation is consistent with an immune-mediated type III hyper-sensitivity reaction to intracameral vancomycin^(7)^.

As the present case illustrates, the natural history of this entity can be visually devastating. Despite adequate treatment with early intravitreal corticosteroids, pan-retinal photocoagulation, and early and repeated intravitreal anti-vascular endothelial growth factor (anti--VEGF) injections, the patient developed neovascular glaucoma one year after presentation, with no improvement in visual acuity.

Due to the rarity of HORV, each surgeon must balance the risk of endophthalmitis and intraocular vancomycin against the potential benefits of intraocular vancomycin prophylaxis. Ophthalmologists should be aware of HORV and, when diagnosed, the affected eye should be aggressively treated with systemic and topical corticosteroids. In some cases, periocular and intravitreous steroids should also be considered. The use of prophylactic intracameral vancomycin in the fellow eye at the time of cataract surgery should also be avoided. Cefuroxime or moxifloxacin can be considered as alter-natives to vancomycin for intracameral prophylaxis.

The patient presented with HORV, which was likely secondary to a hypersensitivity reaction to intracameral vancomycin. HORV is a rare condition that can lead to profound vision loss. Neovascular glaucoma should always be a consideration in HORV cases, as shown in this manuscript.
